# Cerebellar Cortical Neurogenesis following Exposure to Lipopolysaccharide in Ferret Neonates

**DOI:** 10.1159/dne/aclag005

**Published:** 2026-09-07

**Authors:** Shiori Kamiya, Kazuhiko Sawada, Jun Udagawa

**Affiliations:** a Division of Anatomy and Cell Biology, Department of Anatomy, Shiga University of Medical Science, Otsu, Japan; b Department of Nutrition, Koyo Nursing and Nutrition Vocational School, Koga, Japan; c Department of Nutrition, Faculty of Medical and Health Sciences, Tsukuba International University, Tsuchiura, Japan

**Keywords:** Cerebellum, Lipopolysaccharide, Neurogenesis, Ferret, Immunohistochemistry, Bergmann glia, Granular neurons

## Abstract

**Introduction:**

Granular neurons arise from external granular precursors (EGPs) in the external granule layer and internal granular progenitors (IGPs) in the internal granular layer (IGL) during cerebellar cortical histogenesis in ferrets. Additionally, IGPs have a potency on differentiating into Bergmann glia and astrocytes. This study aimed to clarify the effects of lipopolysaccharide (LPS) on granular neurogenesis in the developing ferret cerebellum and its association with the proliferation, differentiation, and apoptosis of EGPs and IGPs.

**Methods:**

LPS (500 μg/g body weight) was administered to ferrets at postnatal days (PDs) 6 and 7. Ferrets also received 5-ethynyl-2′-deoxyuridine (EdU) and 5-bromo-2′-deoxyuridine (BrdU) on PDs 5 and 7, respectively, to label post-proliferative and proliferating cells at the time of LPS exposure.

**Results:**

Two h after BrdU injection (PD 7), the densities of proliferating BrdU- and post-proliferative EdU-labeled cells did not differ in the external granular layer, molecular/Purkinje cell layers (ML/PCL), or IGL between LPS-exposed and control ferrets. BrdU-labeled cells contained 55–83% cells immunostained for proliferating cell nuclear antigen, selectively expressed in IGPs. The S100-immunostained ratio among BrdU-labeled cells was significantly higher in the ML/PCL but lower in the IGL of LPS-exposed ferrets compared to controls. On PD 20, NeuN-positive granular neurons generated on PD 7 (BrdU-labeled) were significantly denser in the IGL of LPS-exposed ferrets.

**Conclusion:**

These findings suggest that LPS enhances granular neurogenesis in the developing cerebellum of ferret neonates. LPS may have a location-specific effect on the glial differentiation of proliferating IGPs: enhancing and reducing in the ML/PCL and IGL, respectively.

## Introduction

The cerebellum regulates muscle tone, integrates sensory and motor functions, and contributes to cognition and emotion [1]. The cerebellar cortex consists of three layers: the molecular layer (ML), the Purkinje cell layer (PCL), and the internal granular layer (IGL). During cerebellar cortical histogenesis, external granular precursors (EGPs) in the external granular layer (EGL) transiently appear and generate granular neurons [2, 3]. Ferrets undergo a cerebellar cortical histogenesis trajectory similar to that of rodents [4, 5], although their development and maturation are relatively slower [5] and exhibit distinct expression patterns of neurogenesis markers such as Pax6 compared with mice [6]. Proliferating cell nuclear antigen (PCNA)-expressing cells transiently appear in the IGL of the developing cerebellum in ferret neonates, and coexpress Nestin, which is selectively expressed in neural stem cells of the central nervous system [7, 8]. Some PCNA-expressing cells were further immunostained with doublecortin (DCX), a marker of immature neurons, and brain fatty acid binding protein and S100, markers of immature and mature glial cells, including Bergmann glia [6]. Thus, PCNA-expressing cells may represent an additional source of granular neurons distinct from EGPs and may also have the potential to differentiate into Bergmann glia and astrocytes as additional sources distinct from their sources known in mice [9]. Based on these characteristics of PCNA-expressing cells, we called them internal granular progenitor (IGP).

Lipopolysaccharide (LPS), an endotoxin located in the outer membrane of Gram-negative bacteria, is an agonist of Toll-like receptor 4 (TLR4) and participates in the innate immune response [10, 11]. LPS stimulates microglia to release pro-inflammatory cytokines such as tumor necrosis factor-alpha and interleukin-6, as well as the anti-inflammatory cytokine interleukin-10, via TLR4 activation [10, 12]. Maternal immune activation (MIA) resulting from maternal bacterial or viral infection increases the risk of neurodevelopmental disorders, including autism spectrum disorder (ASD), schizophrenia, attention deficit/hyperactivity disorder, and cognitive impairment [13–15]. Independent of MIA, bacterial components influence cerebral cortical development through Toll-like receptors, leading to cognitive and behavioral abnormalities [16, 17]. LPS-mediated TLR4 activation regulates adult hippocampal neurogenesis and neurogenesis during brain repair after injury [18–23], promotes the proliferation and differentiation of cultured human neural stem cells, and reduces apoptosis [24]. This study aimed to elucidate the effect of LPS on granular neurogenesis and its association with the proliferation, differentiation, and apoptosis of EGPs and IGPs, two sources of granular neurons identified in the developing ferret cerebellar cortex. Cells generated before LPS exposure were labeled with EdU, whereas cells generated immediately after exposure were labeled with BrdU. This administration strategy enabled independent evaluation of proliferating and post-proliferative EGPs and IGPs in response to LPS.

## Methods

### Animals

The brain samples used in this study were the same as those used in our previous studies investigating the effect of neonatal LPS exposure on the neurogenesis of subventricular zone progenitors on postnatal day (PD) 7 [25] and sulcogyrogenesis on PD 20 [26] in ferrets. The pregnant ferrets were purchased from Japan SLC Co. (Hamamatsu, Japan). Fourteen male ferret offspring obtained via natural delivery from five pregnant ferrets were used in this study. The groups exposed to LPS on PDs 7 and 20 consisted of three and four pups born to different dams, respectively. The control group consisted of littermates of LPS-exposed ferrets at each postnatal age. This approach was used to avoid a litter effect. Ferret infants were reared with lactating dams (3–5 infants per dam) housed in stainless steel cages (80 cm × 50 cm × 35 cm) under 12 h artificial illumination at 21.5 ± 2.5°C at the Faculty of Animal Breeding, Nakaizu Laboratory, Japan SLC. All lactating dams were fed a pellet diet (high density ferret diet 5L14, PMI Feeds, Inc., St. Louis, Mo. USA) and tap water ad libitum. Seven ferret infants received subcutaneous injections of 500 μg/g body weight of LPS on PDs 6 and 7, when the EGL begins to expand in the ferret cerebellar cortex [5]. The remaining infants served as controls. All ferret infants received intraperitoneal injections of 30 μg/g body weight of 5-ethynyl-2′-deoxyuridine (EdU) (Sigma-Aldrich, Missouri, USA) on PD 5 and 30 μg/g body weight of 5-bromo-2′-deoxyuridine (BrdU) (Sigma-Aldrich) on PD 7 to label cells undergoing S phase before and during LPS exposure, respectively. BrdU was administered simultaneously with the second LPS injection on PD 7. Two h after BrdU injection (PD 7), three LPS-exposed and three control ferrets were deeply anesthetized with ∼2% isoflurane inhalation (Fujifilm Wako Pure Chemical Industries, Osaka, Japan) and perfused intracardially with 4% paraformaldehyde (Merck, Darmstadt, Germany) in phosphate-buffered saline (PBS) (pH 7.4). The remaining four LPS-exposed and four control ferrets were reared and perfused with the same fixative on PD 20. The dose of LPS used in this study was shown to enhance the differentiative division of subventricular zone progenitors in ferret neonates in our previous study [25]. The body and brain weights of PD 7 and PD 20 ferrets used in this experiment are shown in online supplementary Table S1 (for all online suppl. material, see https://doi.org/10.1159/dne/aclag005). The body weight in LPS-exposed group was significantly lower than that in controls on PD 7. There was no significant difference in the brain weight between two groups at PDs 7 and 20.

### Preparation of Tissue Sections

The cerebellum was immersed in 30% sucrose-PBS solution overnight and embedded in optimal cutting temperature compound at −70°C (Sakura Fine Tech Japan, Tokyo, Japan). After embedding, 100-μm-thick sagittal cryosections of the cerebellar vermis were prepared using a Retratome (REM-700: Daiwa Koki) equipped with an electro freezer (MC-802A, Daiwa Koki, Japan). All sections were collected in vials containing 4% paraformaldehyde-PBS.

### Immunohistochemical Procedures

All immunohistochemical procedures were performed on floating sections. Antigen retrieval was conducted by heating sections in Antigen Retrieval Reagent UNIVERSAL (R&B system, Minneapolis, MN, USA) at 90°C for 30 min in a water bath. After cooling, sections were incubated in 0.1% Triton X-100-PBS at 37°C for 1 h, followed by treatment with the Click-iT EdU reaction cocktail for EdU detection and subsequent immunofluorescence staining using the primary and secondary antibodies listed in Tables S2 and S3. The primary antibodies were highly specific for ferret brain tissue [6, 9, 26]. The terms “positive labeling” or “immunopositive” (+) refer to cells exhibiting high to moderate levels of thymidine analog labeling or marker antigen immunostaining.

### Evaluation of Cell Density

A 10-μm-thick digital image stack composed of 10 sections at 1 μm intervals along the Z-axis from the most superficial plane was acquired using an AxioImager M2 ApoTome.2 with a 20× objective lens, equipped with an AxioCam MRm camera (Zeiss, Gottingen, Germany) and Zen 2.3 blue edition software (Zeiss). Two sectional images, 4 μm apart in the Z-direction (third and seventh from the superficial slices), were selected as the reference and lookup images, respectively. The density of thymidine analog-labeled or immunostained cells was estimated using the disector method with systematic random sampling following a previously established procedure [9, 27]. Measurements were performed in the lobules III, IV, V, VI, VIII, and ventral IX of the cerebellar vermis, and the mean value was used as the individual value. In each section, six square counting frames (40 × 40 μm) were used to systematically select regions of interest (ROIs) randomly positioned in the EGL, molecular/Purkinje cell layers (ML/PCL), and IGL in PD 7 ferrets, or in the ML, PCL, and IGL in PD 21 ferrets, in both the reference and lookup images at corresponding positions perpendicular to the surface. Thymidine analog-labeled, or immunostained cells were counted within the ROIs using the “forbidden line” rule. The densities were calculated using the following formula: [Cell density = Qn−/(a × b × t)] (Qn− = total number of thymidine analog-labeled and/or immunostained cells appearing within ROIs in the lookup images but not in the reference images; a = 6, total number of ROIs per animal; b = 40 μm × 40 μm area of the counting frame; and t = 4 μm, distance between the lookup and reference images). Densities of thymidine analog-labeled or immunostained cells in each cortical layer were estimated by summing measurements. To address the post-proliferative (EdU-labeled) and proliferating (BrdU-labeled) cell types at the time of LPS exposure, we calculated the antigen-positive ratios for each immunostained cell among thymidine analog-labeled cells in the cerebellar cortical layers on PD 7 and expressed the data as the mean ± standard deviation of individual values. Because percentages were calculated independently for each marker antigen, overlap between markers could not be determined.

### Statistical Analysis

Densities of thymidine analog-labeled, immunolabeled cells and the antigen-positive ratios for each immunostained cell among thymidine analog-labeled cells were analyzed using repeated-measures two-way analysis of variance (ANOVA), with cerebellar cortical layer (PD 7: EGL, ML/PCL, and IGL; PD 20: ML, PCL, and IGL) and group (LPS-exposed and control ferrets) as factors. Post hoc comparisons were performed using the Tukey test, with *p* < 0.05 considered statistically significant.

## Results

### Distribution and Density of EdU+ and BrdU+ Cells in the Cerebellar Cortex of PD 7 Ferrets

Cells undergoing S phase 24 h before LPS administration (PD 5) were labeled with EdU, whereas cells undergoing S phase immediately after LPS exposure (PD 7) were labeled with BrdU. EdU+ and BrdU+ cells were observed in all layers of the cerebellar cortex in PD 7 ferrets; EdU+ cells were densely aligned in the inner zone of the EGL (Fig. 1a). Figure 1b shows the density of EdU+ and BrdU+ cells in each cortical layer. No differences were detected in the densities of EdU+ or BrdU+ cells in any cortical layers between LPS-exposed and control ferrets.

**Fig. 1.**
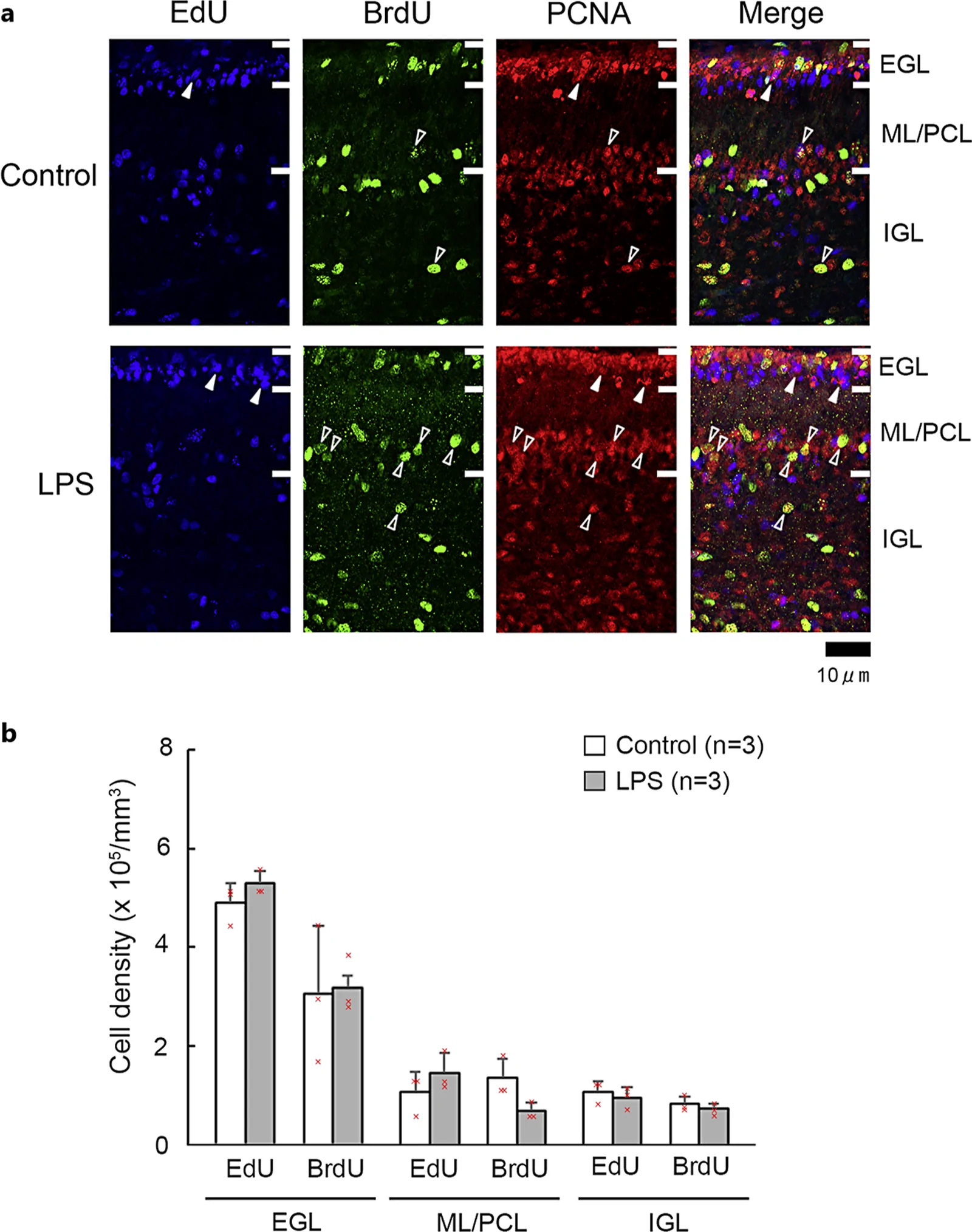
Immunofluorescence for PCNA with EdU and BrdU labeling in the cerebellar cortex of LPS-exposed and control ferrets at PD 7. **a** Representative immunofluorescence images showing PCNA staining with EdU and BrdU labeling. Closed arrowheads indicate EdU-labeled PCNA-immunopositive cells. Open arrowheads indicate BrdU-labeled PCNA-immunopositive cells. **b** Bar graph showing densities of EdU- and BrdU-labeled cells in cerebellar cortical layers. Data are presented as mean ± standard deviation, and individual data points are overlaid. EGL, external granular layer; IGL, internal granular layer; ML/PCL, molecular and Purkinje cell layers.

### Immunostained Ratio of Neurogenesis and Apoptosis Markers in Thymidine Analog-Labeled Cells in the Cerebellar Cortex of PD 7 Ferrets

In addition to EdU and BrdU labeling, multiple immunofluorescence staining was performed using antibodies against PCNA, DCX (a marker of immature neurons), NeuN (a marker of granular neurons), S100 (a marker of glial cells), and cleaved caspase 3 (a marker of apoptosis). We addressed the cell types of post-proliferative (EdU-labeled) and proliferating (BrdU-labeled) at the time of LPS exposure. Here, PCNA+ cells distributed in the ML/PCL and IGL were defined as IGPs, because PCNA is selectively expressed in IGPs in these cortical layers of ferret neonates [6]. Because only a small number of EdU+/BrdU+ cells were observed in the cerebellum of both LPS-exposed and control ferrets, the proportion of immunolabeling for each antigen (PCNA, DCX, NeuN, S100, and cleaved caspase 3) in EdU+/BrdU+ cells was not evaluated.

PCNA immunostaining was found in cells densely aligned in the EGL, which were defined as EGPs, in both groups on PD 7 (Fig. 1a). The proportion of PCNA+ cells was 10–26% in EdU+ EGPs and 26–39% in BrdU+ EGPs, which were not different between LPS-exposed and control ferrets (Fig. 2a). PCNA + cells, defined as IGPs, were abundant in the ML/PCL and upper IGL (Fig. 1a). More than 55% of BrdU+ cells on average were PCNA-positive without significant difference between two groups of ferrets, while the PCNA+ ratio was accounted for at 83.4 ± 2.3% in the ML/IGL of LPS-exposed ferrets (Fig. 1a, 2b). In contrast, PCNA+ cells among EdU+ cells were retained at 18–26% in this cortical layer of LPS-exposed and control ferrets (Fig. 1; 2a).

**Fig. 2.**
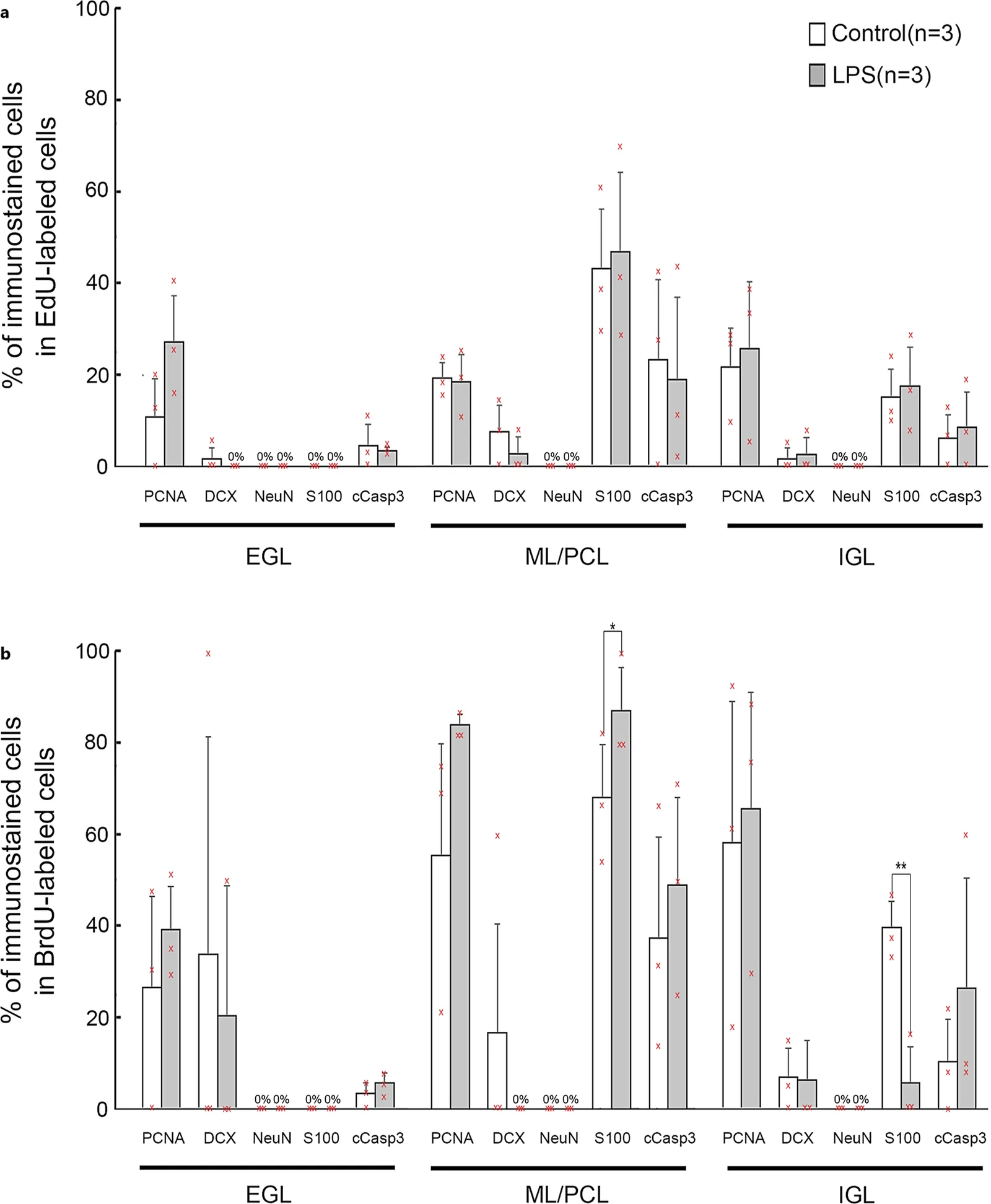
Bar graphs showing percentages of immunostained cells for various markers among EdU- and BrdU-labeled cells in cerebellar cortical layers of LPS-exposed and control ferrets at PD 7. **a** Percentages of immunostained cells for various markers in EdU+ cells. **b** Percentages of immunostained cells for various markers in BrdU+ cells. Data are presented as mean ± standard deviation, and individual data points are overlaid. EGL, external granular layer; IGL, internal granular layer; ML/PCL, molecular and Purkinje cell layers.

A few or no EdU+ or BrdU+ cells were DCX+ in the EGL, ML/PCL and IGL of LPS-exposed and control ferrets on PD 7 (Fig. 3a). Furthermore, neither EdU+ nor BrdU+ cell was NeuN-negative in any cortical layers examined in both two groups (Fig. 3b). Thus, post-proliferative and proliferating cells, including EGPs in the EGL and IGPs in the ML/PCL and IGL, at the time of LPS exposure may not undergo terminal differentiation into granular neurons (Fig. 3b).

**Fig. 3.**
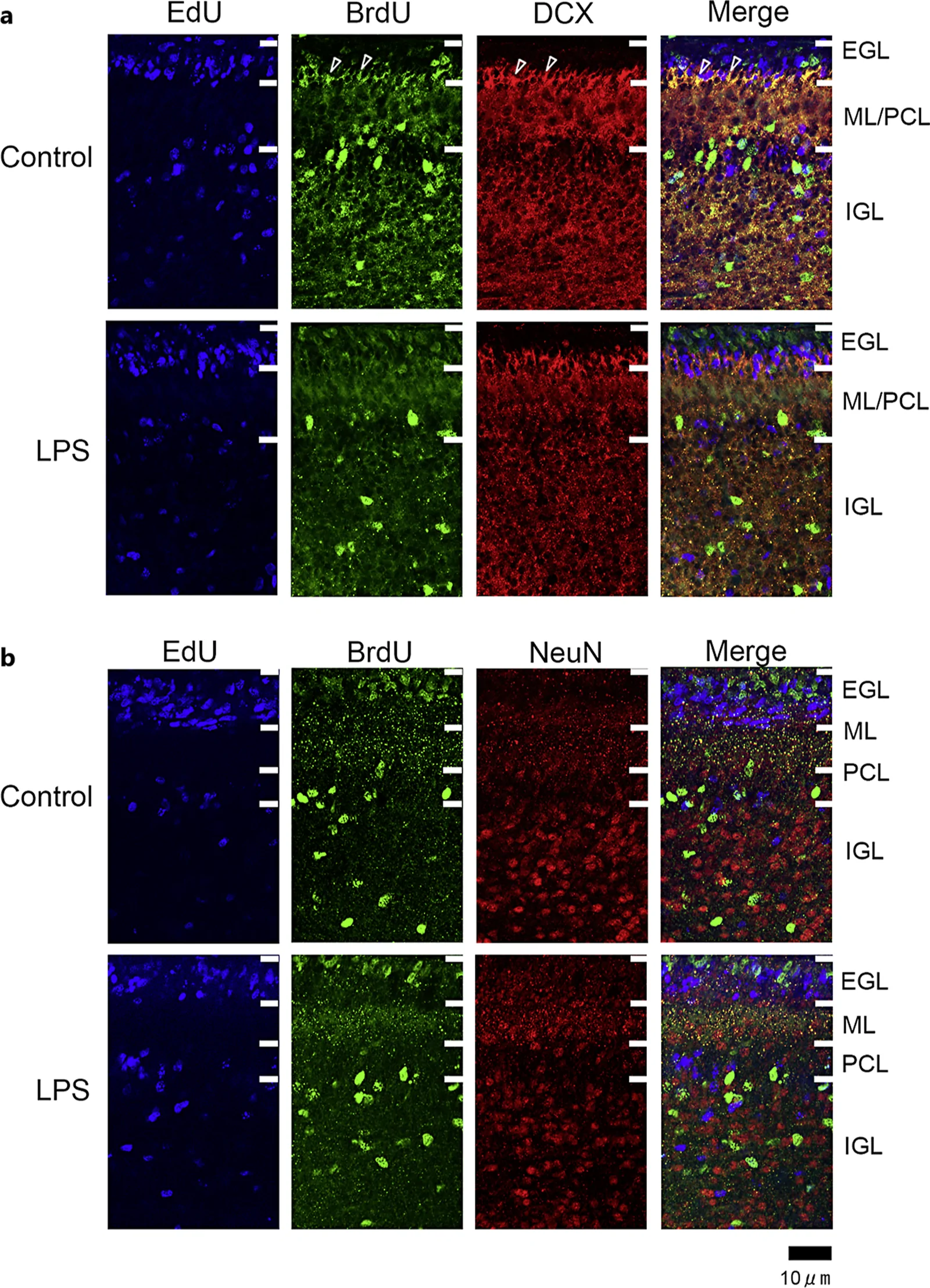
Immunofluorescence for DCX or NeuN with EdU and BrdU labeling in the cerebellar cortex of LPS-exposed and control ferrets at PD 7. **a** Representative immunofluorescence images showing DCX staining with EdU and BrdU labeling. Open arrowheads indicate BrdU-labeled DCX-immunopositive cells. **b** Representative immunofluorescence images showing NeuN staining with EdU and BrdU labeling. EGL, external granular layer; IGL, internal granular layer; ML/PCL, molecular and Purkinje cell layers.

S100 immunoreactivity has been observed in glial cells, such as Bergmann glia in the PCL and upper IGL, astrocytes in the lower IGL, and PCNA+ IGPs differentiating into glial cell lineages in the cerebellum of ferret neonates [9]. In the present study, S100+ cells were abundant in the ML/PCL and sparse in the IGL of LPS-exposed and control ferret cerebella on PD 7 (Fig. 4a). A cortical layer-related change in the proportion of S100+ cells among BrdU+ cells was revealed by repeated-measures two-way ANOVA-defined significant interaction between the cerebellar cortical layer and group [*F*_(1,4)_ = 10.829; *p* < 0.01]. Tukey’s post hoc analysis indicated that the percentage of S100+ cells among BrdU+ cells was significantly higher in the ML/PCL (*p* < 0.05) but significantly lower in the IGL (*p* < 0.01) of LPS-exposed ferrets than in controls (Fig. 2a). These results suggest that LPS affects the glial differentiation of proliferating IGPs with the effect depending on their location: enhanced in the ML/PCL but reduced in the IGL.

**Fig. 4.**
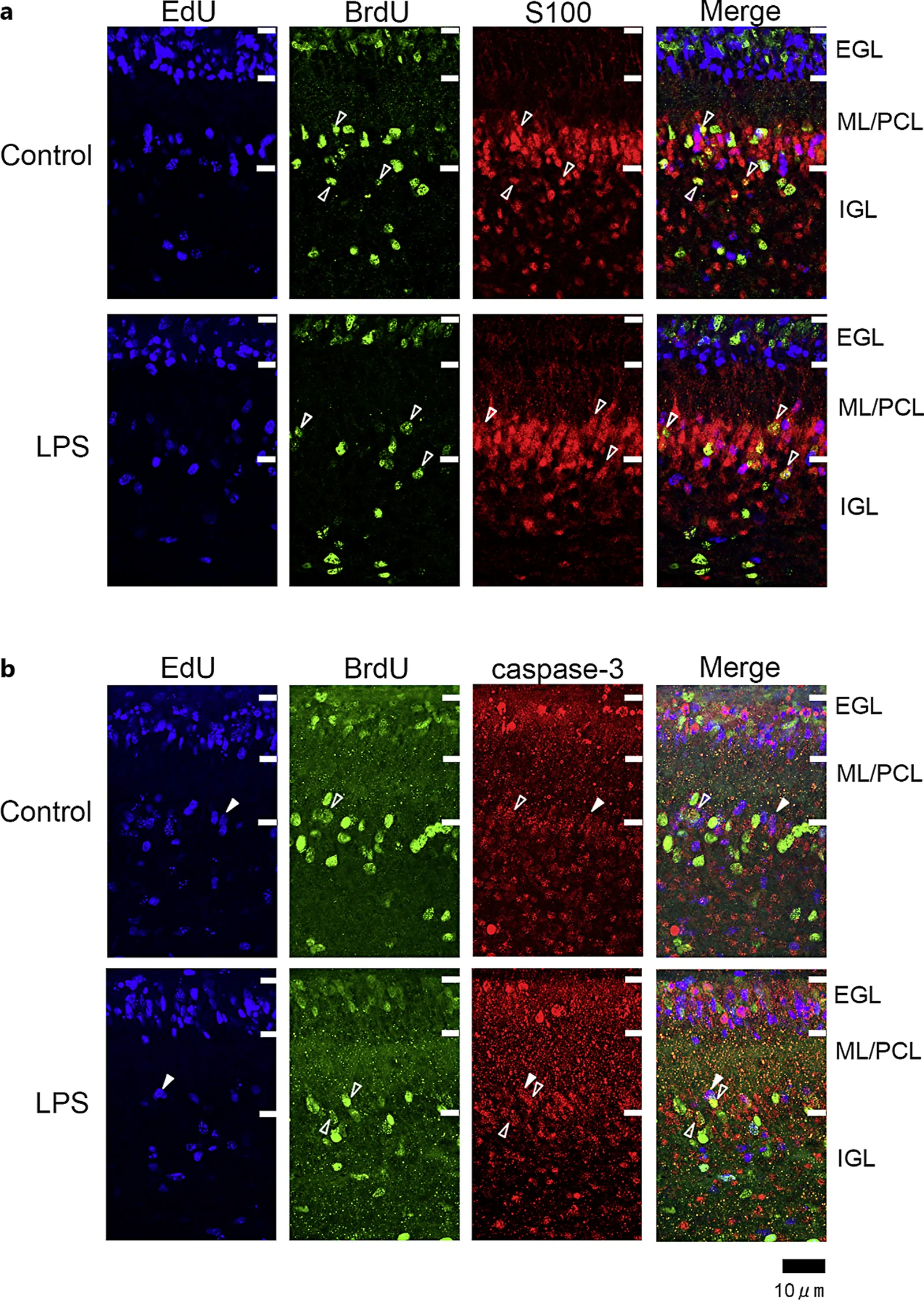
Immunofluorescence for S100 or cleaved caspase 3 with EdU and BrdU labeling in the cerebellar cortex of LPS-exposed and control ferrets at PD 7. **a** Representative immunofluorescence images showing S100 staining with EdU and BrdU labeling. Open arrowheads indicate BrdU-labeled S100-immunopositive cells. **b** Representative immunofluorescence images showing cleaved caspase 3 staining with EdU and BrdU labeling. Closed arrowheads indicate EdU-labeled cleaved caspase 3-immunopositive cells; open arrowheads indicate BrdU-labeled, cleaved caspase 3-immunopositive cells. EGL, external granular layer; IGL, internal granular layer; ML/PCL, molecular and Purkinje cell layers.

Cleaved caspase 3+ cells were distributed across all cortical layers examined in the cerebellar cortex of PD7 ferrets (Fig. 4b). There was no difference in the proportion of cleaved caspase 3+ cells among EdU+ and BrdU+ cells in each cortical layer (Fig. 2).

### Densities of Cells Immunostained with Neurogenesis and Apoptosis Markers in the Cerebellar Cortex of PD 7 Ferrets

The density of cells immunostained for neurogenesis and apoptosis markers was estimated in the cerebellar cortex of ferret neonates at PD 7. A cortical layer-related change in the density of cleaved caspase 3+ cells was revealed by repeated-measures two-way ANOVA-defined significant interaction between the cerebellar cortical layers and the groups [*F*_(*1*,*4*)_ = 13.000; *p* < 0.01]. Tukey’s post hoc analysis indicated a significantly higher density of cleaved caspase 3+ cells in the ML/PCL of LPS-exposed ferrets than in controls (*p* < 0.01) (Fig. 5). These results suggest that apoptosis is increased in cells, particularly in the ML/PCL. As mentioned above, post-proliferative (EdU+) and proliferating (BrdU+) cells in the ML/PCL included cleaved caspase 3+ cells at approximately 20% and 50%, respectively, without differences between LPS-exposed and control ferrets (Fig. 2). Therefore, LPS may increase the apoptosis of cells located particularly in the ML/PCL of the PD 7 ferret cerebellum, independent of their proliferative status, although the cell types could not be identified. In contrast, no differences were detected in the densities of cells immunostained for other antigens, including PCNA, DCX, NeuN, and S100, across all three cortical layers between the LPS-exposed and control ferrets (Fig. 5).

**Fig. 5.**
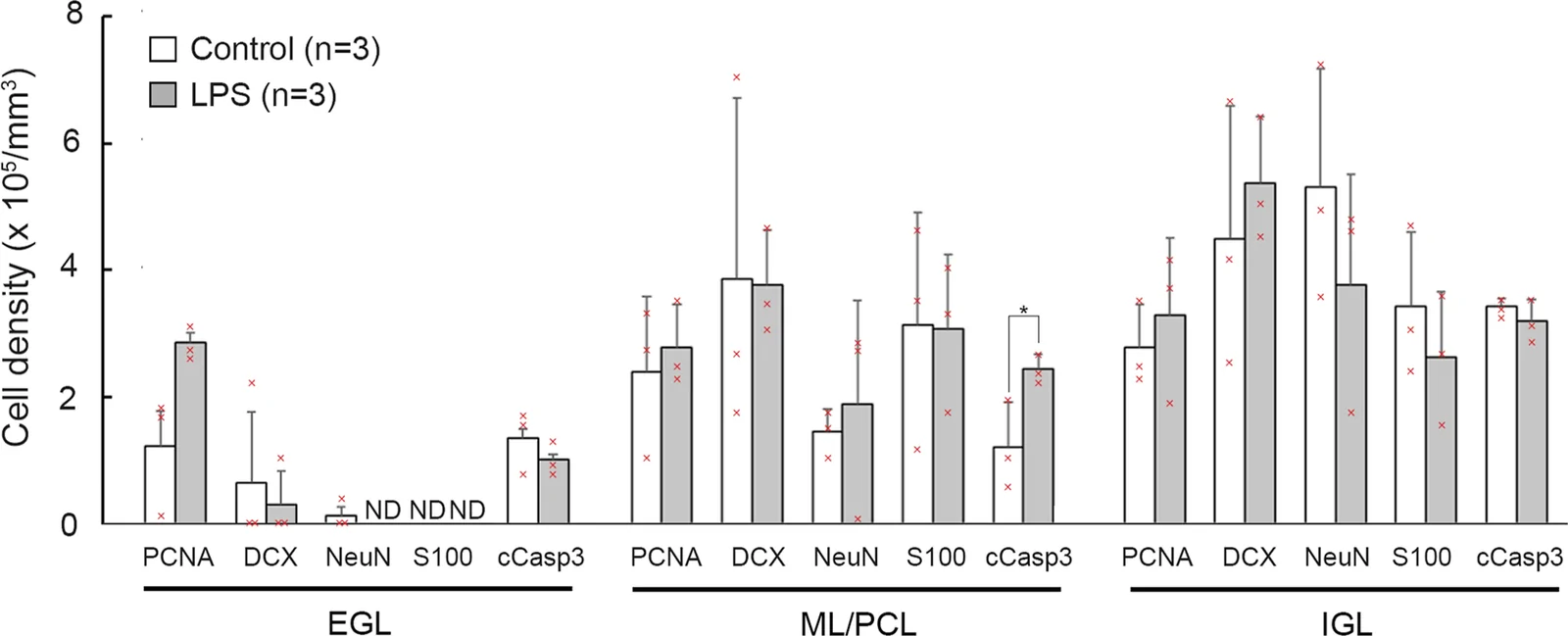
Bar graphs showing densities of cells immunostained for various markers in the cerebellar cortex of LPS-exposed and control ferrets at PD 7. Data are presented as mean ± standard deviation, and individual data points are overlaid. Significant differences were detected by Tukey’s post hoc test at *p < 0.01. IGL, internal granular layer; ND, not detected; ML, molecular layer; PCL, Purkinje cell layer.

### Densities of Granular Neurons and Glial Cells in the Cerebellar Cortex of PD 20 Ferrets

Figure 6a shows the images of NeuN+ granular neurons generated at PD 5 (EdU+) and PD 7 (BrdU+) in the cerebellar cortex of PD 20 ferrets. No significant difference was observed between LPS-exposed and control groups in the density of EdU+/NeuN+ granular neurons across all three cortical layers (Fig. 7a). A significant interaction between the cerebellar cortical layers and the groups was detected in the density of BrdU+/NeuN+ granular neurons by repeated-measures two-way ANOVA [*F*_(1,6)_ = 14.013; *p* < 0.01]. Tukey’s post hoc analysis revealed a significantly higher density of BrdU+/NeuN+ granular neurons in the IGL of LPS-exposed ferrets than in controls (*p* < 0.01) (Fig. 7a). Thus, granular neurons, which were generated on PD 7 immediately following LPS exposure, showed the increased density in the IGL at PD 20. S100+ cells were abundant in the PCL and sparse in the IGL (Fig. 6b). There was no difference in the densities of EdU+ or BrdU+ S100+ cells in all three cortical layers examined between LPS-exposed and control ferrets (Fig. 7b). In addition, no EdU+ or BrdU+ Purkinje cells (parvalbumin+/Calbindin D28k+) or molecular layer interneurons (MLI; parvalbumin+/Calbindin D28k-negative) were observed in the cerebellar cortex of PD 20 ferrets (online suppl. Fig. S1), indicating that neural progenitors, including IGPs, EGPs, and MLI progenitors proliferating on PDs 5 and 7, did not differentiate into Purkinje cells or MLIs following LPS exposure.

**Fig. 6.**
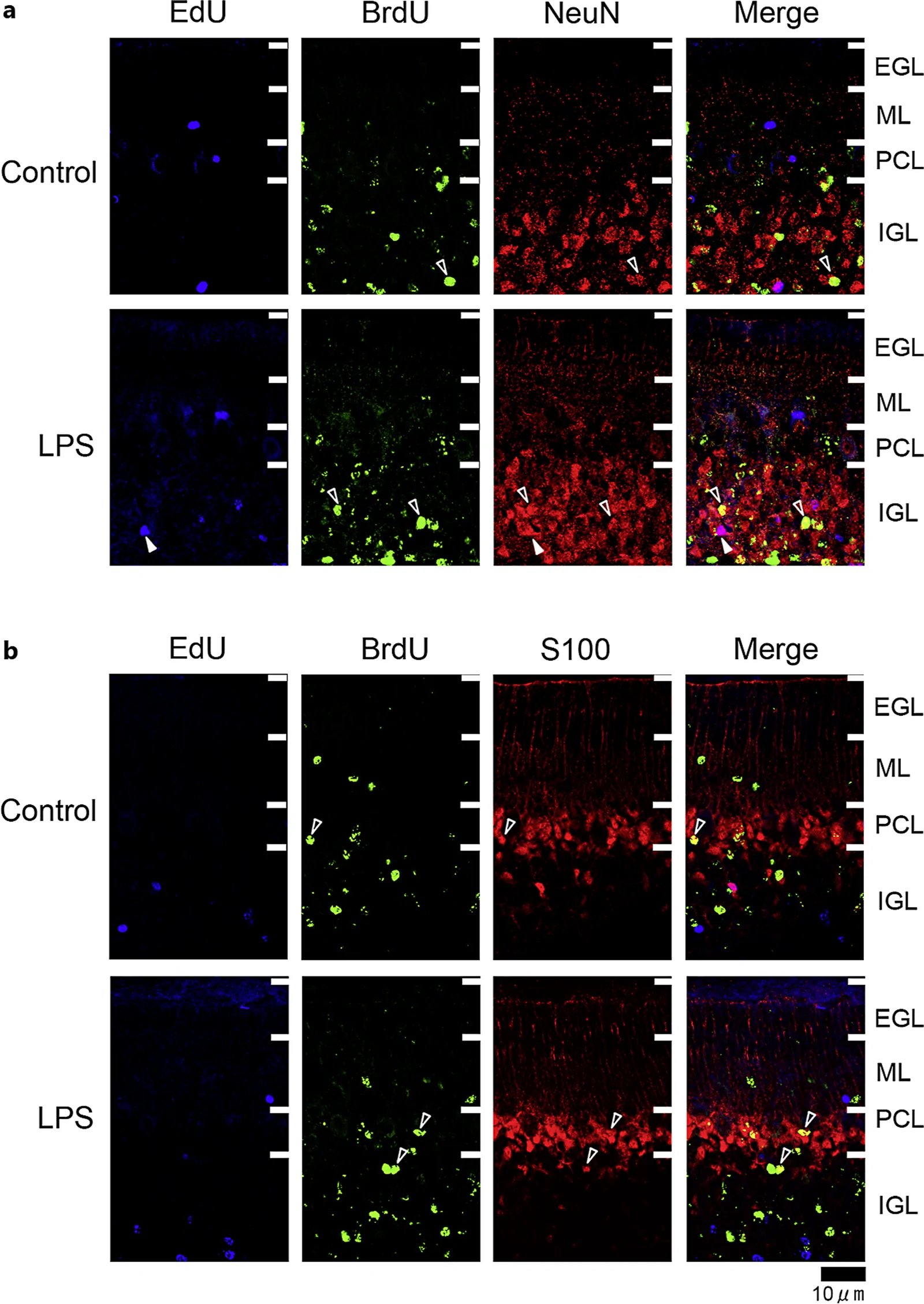
Immunofluorescence for NeuN or S100 with EdU and BrdU labeling in the cerebellar cortex of LPS-exposed and control ferrets at PD 20. **a** Representative immunofluorescence images showing NeuN staining with EdU and BrdU labeling. Closed arrows indicate EdU-labeled NeuN-immunopositive cells; open arrows indicate BrdU-labeled NeuN-immunopositive cells. **b** Representative immunofluorescence images showing S100 staining with EdU and BrdU labeling. Open arrows indicate BrdU-labeled S100-immunopositive cells. EGL, external granular layer; IGL, internal granular layer; ML/PCL, molecular and Purkinje cell layers.

**Fig. 7.**
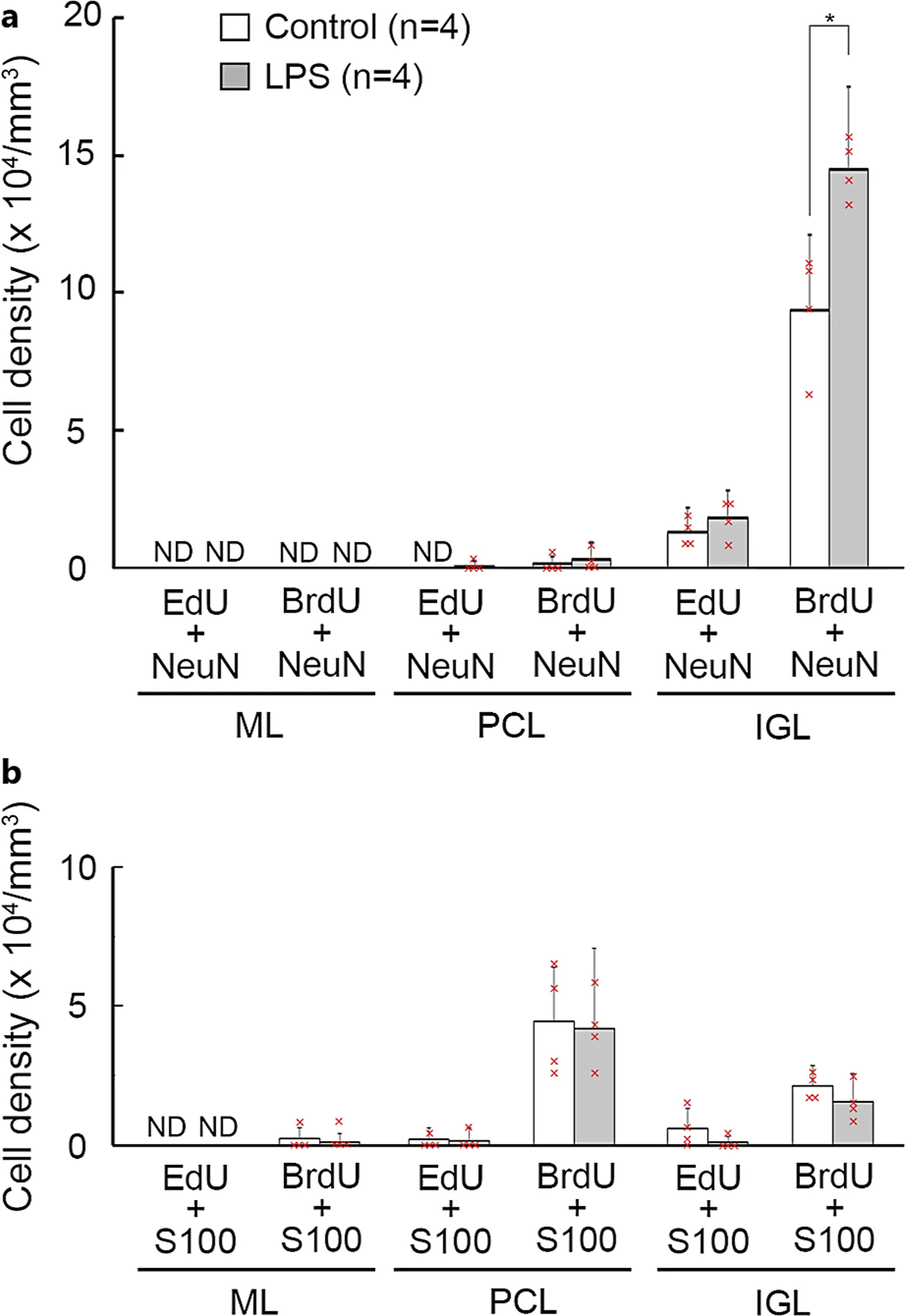
Bar graphs showing densities of EdU- or BrdU-labeled granular neurons and glial cells in the cerebellar cortex of LPS-treated and control ferrets at PD 20. **a** Density of EdU- or BrdU-labeled NeuN-immunopositive cells. **b** Density of EdU- or BrdU-labeled S100-immunopositive cells. Data are presented as mean ± standard deviation, and individual data points are overlaid. Significant differences were detected by Tukey’s post hoc test at *p < 0.01. ML, molecular layer; IGL, internal granular layer; PCL, Purkinje cell layer.

## Discussion

Like polyinosinic: polycytidylic acid (Poly I: C), LPS is used to generate MIA models that exhibit ASD- and schizophrenia-like behavioral abnormalities, altered histoarchitecture, and hypomyelination of the cerebral cortex [28–31]. LPS is a natural agonist of TLR4 [10, 11] with the capacity to modulate proliferation, differentiation, and apoptosis of neural stem cells/progenitors [24]. In our recent study, neonatal LPS exposure in ferrets enhanced differentiative division of subventricular zone progenitors into cerebral cortical neurons [25], resulting in altered sulcal infolding [26], a feature frequently observed in neurodevelopmental disorders such as ASD and schizophrenia [32, 33]. The present investigation attempted to elucidate the effect of LPS on granular neurogenesis, particularly focusing on EGPs and IGPs, two sources of granular neurons identified in the ferret cerebellar cortex [6, 9, 26]. The findings revealed that LPS induced granular neurogenesis. However, this did not change cerebellar cortical volume [26]. Granular neurons provide excitatory mossy fiber input to Purkinje cells, which is inhibitory regulated by MLIs [2]. In this study, LPS exposure did not induce MLI neurogenesis. Therefore, the excess production of granular neurons following neonatal LPS exposure may disrupt the balance between the excitatory and inhibitory inputs that regulate Purkinje cell activity, indicating developmental alterations relevant to neurodevelopmental disorders.

In this study, LPS also affected glial differentiation in addition to granular neurogenesis. Bergmann glia are derived from radial glia in the ventricular zone of the 4th ventricle roof of the embryonic cerebella and extend radial processes at an immature stage [34]. Immature Bergmann glia relocate and align in the PCL and subsequently differentiate into mature Bergmann glia during the early postanal age in mice [35]. A population of astrocytes in the cerebellar IGL emerges from immature Bergmann glia in mice [35]. Recently, we reported that IGPs expressing Nestin and PCNA transiently appear in the IGL of the developing cerebellum in ferret neonates [6] and suggested that IGPs have multipotency with differentiation into glial cells, such as Bergmann glia and astrocytes, in addition to granular neurons in response to VPA [9, 26]. IGPs extend the BLBP+ radial processes through the ML to the EGL, forming a Bergmann glia-like morphology [9]. Therefore, IGPs may represent a subpopulation of immature Bergmann glia that arise from the 4th ventricle ventricular zone. A notable finding of this study was that glial differentiation of BrdU+ proliferating cells, which contained PCNA+ cells defined as IGPs at 55–83% in the ML/PCL and IGL of the PD 7 ferret cerebellum, was enhanced in the ML/PCL but reduced in the IGL following LPS exposure. Such a difference in the effect of LPS on the glial differentiation of proliferating IGPs may be involved in cerebellar cortical layer-related microenvironmental influences, for example, sonic hedgehog, which is secreted from Purkinje cells and is required for Bergmann glial development during early postnatal ages [36]. Although this study could not elucidate the sources of granular neurons produced following LPS exposure, the IGL-specific reduction in glial differentiation predicts that LPS redirects IGP differentiation towards non-glial cells such as granular neurons.

LPS-mediated TLR4 activation enhances proliferation and neuronal differentiation of cultured human neural stem cells and reduces apoptosis [24]. Apoptosis of hippocampal dentate gyrus progenitors is also induced by LPS exposure in ferret neonates [37]. TLR4 activation-induced apoptosis has been reported in microglia [38] but not in other glial cells. Thus, LPS/TLR4 signal-modulated apoptosis may diverse among types of neuronal and/or glial cells. In the current study, LPS increased the apoptosis of cells located particularly in the ML/PCL in the ferret cerebellum on PD 7, independent of their proliferative status, although the cell types could not be identified. Further studies are needed to identify the types of apoptotic cells to understand the effects of LPS on the developing cerebellum in relation to the pathogenesis of neonatal LPS-triggered neurodevelopmental disorders.

The findings of this study are summarized in Figure 8. This study attempted to elucidate the effect of LPS on granular neurogenesis and its association with the proliferation, differentiation, and apoptosis of two types of granular neurons, EGPs and IGPs. Neonatal exposure to LPS may affect the glial differentiation of proliferating IGPs, depending on their cortical location, and enhance granular neurogenesis. IGPs, one of the targets of LPS in this study, have been found in ferrets [5, 6] but not yet in mice. Ferrets have disadvantageous characteristics for using neurodevelopmental studies that a sufficient number of ferrets are not easy to correct because of seasonal breeding and small litter sizes. Therefore, the sample size used in this study was small (*n* = 3 or 4). Future studies with larger cohorts are required to substantiate the findings of this study.

**Fig. 8.**
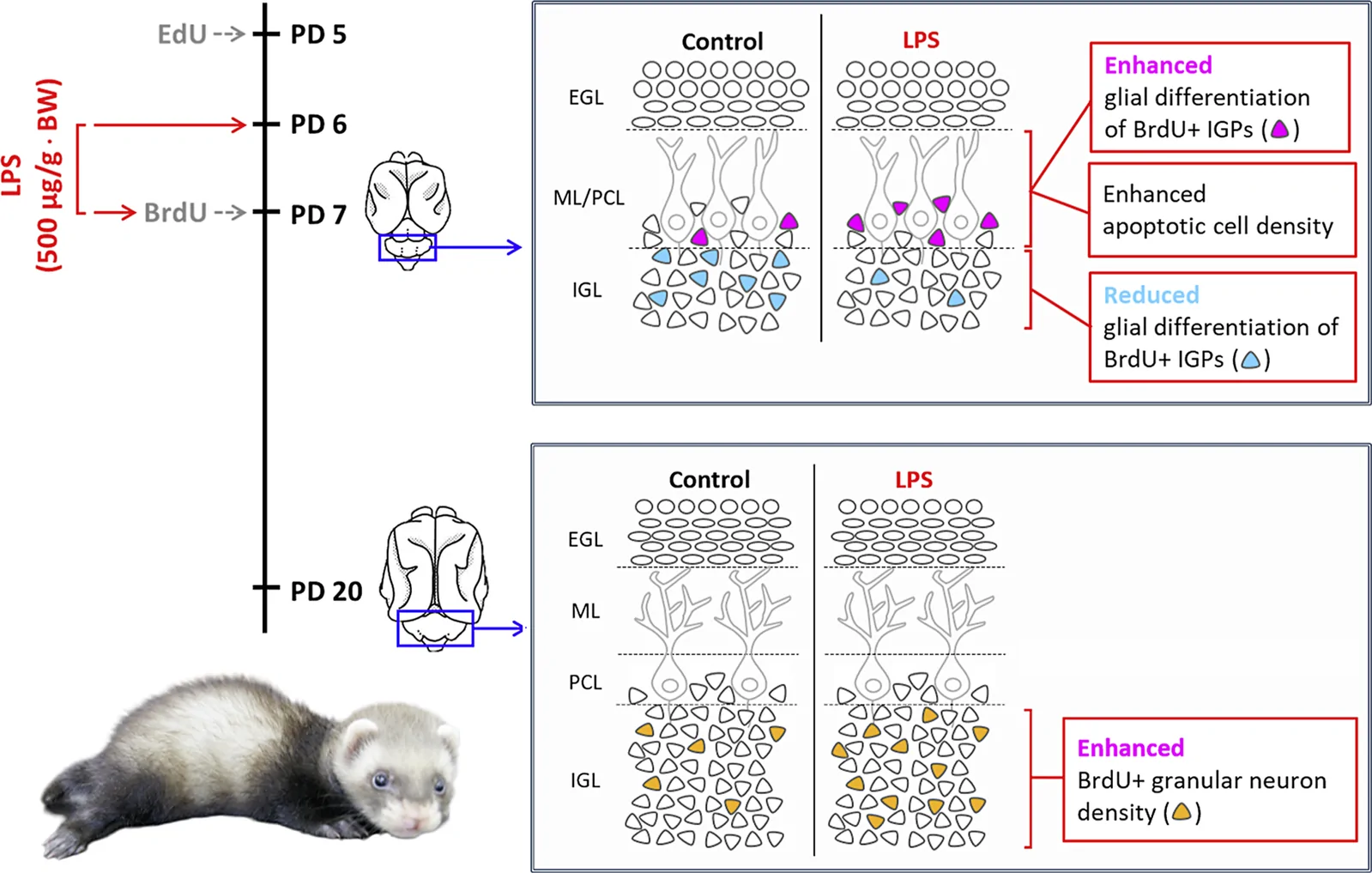
Schematic summary figure of the effect of neonatal LPS exposure on the cerebellar cortex of ferrets. On PD (PD) 7, ≥ 55% of BrdU-labeling was obtained in PCNA-immunopositive internal granular progenitors (IGPs). Neonatal LPS exposure enhanced the proportion of S100 immunostaining among BrdU-labeled cells in the ML/PCL (magenta) but reduced it in the IGL (cyan) on PD 7. On PD 20, the density of BrdU-labeled NeuN-immunopositive granular neurons (orange) increased in the IGL of LPS-exposed ferrets. Non-colored cells other than Purkinje cells in the PCL on both PDs 7 and 20 represent Bergmann glia or IGPs. EGL, external granular layer; IGL, internal granular layer; ML, molecular layer; PCL, Purkinje cell layer.

## Statement of Ethics

This study was approved by the Institutional Animal Care and Use Committee of Tsukuba International University (Approval No. 30–1, December 4, 2018). Every effort was made to minimize the number and suffering of animals used in this study.

## Conflict of Interest Statement

The authors declare no conflicts of interest.

## Funding Sources

This work was partly supported by the Japan Society for the Promotion of Science (JSPS) KAKENHI (Grant No. 21K06741, awarded to K.S.).

## Author Contributions

Conceptualization, formal analysis, investigation, methodology, validation, and writing – original draft: Shiori Kamiya. Supervision and writing – review and editing: Jun Udagawa. Conceptualization, funding acquisition, project administration, and writing – review and editing: Kazuhiko Sawada.

## Data Availability

The original contributions of this study are included in the article and online supplementary material. Further inquiries can be directed to the corresponding author.
